# Editorial: Enhancing quality of life in individuals with neurodevelopmental disorders: pathways to inclusion and well-being

**DOI:** 10.3389/fpsyt.2026.1891617

**Published:** 2026-06-19

**Authors:** Antonio M. Amor, Cristina Mumbardó-Adam, Miguel Á. Verdugo, Luigi Croce

**Affiliations:** 1 Department of Personality, Assessment, and Psychological Treatments. University of Salamanca, Salamanca, Spain; 2 Institute for Community Inclusion. University of Salamanca, Salamanca, Spain; 3 Department of Cognition, Development and Educational Psychology, Universitat de Barcelona, Barcelona, Spain; 4 Centro Domino per l’Autismo, Milan, Italy

**Keywords:** inclusion, input-throughput-outcome, neurodevelopmental disorders (NDD), outcome, quality of life, rights

## Introduction and overview

Over the last decades, there has been a paradigm shift regarding persons with neurodevelopmental disorders (NDD): deficit-based approaches have been superseded by a socio-ecological paradigm. This framework focuses on the relationship between the person and the environment in which they live; the understanding of their holistic support needs required to participate in self-determined life projects; the recognition of personal strengths beyond support needs; and, ultimately, the promotion of their inclusion across all societal strata and throughout the lifespan (1, 2).

Within this framework, multidimensional individual quality of life (QoL) approaches are essential. These models focus on the person, moving beyond a strictly physical or emotional health perspective to encompass other vital life domains, as well as the person’s own lived experience rather than merely objective living conditions (see, e.g (3–5). The importance of adopting QoL approaches within this paradigm of NDD lies in the fact that, as an *input* variable, QoL reveals what is important to the person. This insight lends meaning to all subsequent interventions aimed at enhancing the well-being and inclusion of people living with these conditions, including the co-development of life projects, as well as support assessment and planning (6). Furthermore, QoL is also well-suited to be evaluated as an *outcome* variable, allowing for an assessment of the impact that implemented interventions—designed to achieve the person’s life project—have had on their life (7). Indeed, certain frameworks, such as the one proposed by Schalock and Verdugo (5), provide indicators that are sensitive to the rights enshrined in the United Nations Convention on the Rights of Persons with Disabilities (8). Consequently, a person’s QoL can reflect their satisfaction with, as well as their access to and participation in, areas that represent key rights and, therefore, true inclusion (6, 9).

These frameworks provide the foundation for the Research Topic featured in this editorial. The purpose of this editorial is to analyze the contributions made by the authors through a QoL lens, emphasizing whether their studies consider QoL or related concepts (e.g., resilience, self-determination) as an outcome variable, or if they adopt a vision that views QoL (or associated constructs) as a driving force for inclusion-oriented actions (i.e., as an input variable upon which to build support strategies that will be subsequently evaluated).

## Contributions addressing quality of life as an *outcome* measure

Ten studies addressed QoL (or related constructs) as an outcome variable. Two of these explored QoL through a qualitative approach. Ramsdal et al. analyzed interpersonal relationships and emotional well-being—both core domains of QoL—as outcome variables to validate intervention efficacy. In their study, which provided competitive integrated employment opportunities to five adults with intellectual disability (ID), participants reported a strong sense of belonging in the workplace, signaling the success of the intervention. Similarly, Aguayo et al. conducted an interpretive study on independent living among adults with cerebral palsy in Spain, interviewing 165 family caregivers. Four overarching themes emerged from the interviews: independence, supports, family care, and well-being. Regarding well-being, the findings underscored the urgent need for structural changes to foster a meaningful improvement in the lives of this population.

Three papers utilized a comparative design. Sforza et al. compared QoL and disease burden (as dependent variables) between two groups: 66 children with tuberous sclerosis complex (TSC) and 63 children with idiopathic autism spectrum disorder (ASD). Their study revealed a lower overall QoL in children with syndromic autism and other conditions, such as epilepsy (i.e., the TSC group), compared to those with idiopathic ASD. For their part, Dong et al. compared the QoL of 217 parents of children with ID against that of the general population. They found significantly lower outcomes in the former group, whose QoL was compromised by emotional distress and a lack of resources. Finally, Gómez-Vela et al. compared school participation—a construct closely linked to QoL—between students with (*n* = 142) and without disabilities (*n* = 273) in Spain, utilizing perspectives provided by their families. The results revealed lower rates of participation and engagement across all school activities within the group of students with disabilities.

Two studies employed a predictive design. Wurth et al. analyzed how diagnosis acceptance, masking, and perceived benefits and challenges predicted QoL in a sample of 1,056 adults with ADHD and ASD. QoL was significantly associated with these variables, with perceived benefits demonstrating the strongest association. Shaw et al. conducted a logistic regression analysis within a sample of 272 autistic Americans. Their findings revealed that goal-setting and psychological empowerment universally predicted all studied QoL-related outcomes (i.e., moving into their own home, falling in love, getting married, pregnancy, becoming a caregiver, and being arrested). Additionally, autonomy, decision-making, and social skills predicted specific milestones, such as housing and romance, while a larger support network significantly increased the likelihood of finding romance.

Other studies did not fall into the aforementioned categories. Through an online survey administered to 347 Hungarian parents, Kárpáti and Miklósi explored how parents conceptualize different types of disabilities and how these perceptions influence children’s educational inclusion and social participation (as outcome variables). Utilizing network analysis, they identified a triadic structure that explains inclusion—comprising parental knowledge, self-efficacy toward inclusion, and willingness to include. Their findings revealed gaps between the willingness to include and perceived self-efficacy, as well as less favorable attitudes toward inclusion regarding ID, ADHD, and language disorders. In their mini-review, Fernández et al. highlighted the challenges of assessing QoL in autistic adults with lower support needs, emphasizing essential components for designing reliable, valid, and culturally adapted tools. Finally, Sánchez-Gómez et al. conducted a cross-sectional descriptive study analyzing restrictive practices across 45 disability support organizations. Although the occurrence of these practices was low, the most frequent ones (i.e., leisure limitations and inflexible mealtimes) pose a direct threat to the QoL of people with NDD.

## Contributions addressing quality of life as an *input* and catalyst for inclusive practice

These studies conceptualize QoL or related constructs as a baseline for designing interventions within a logic model that adopts an input-throughput-outcome scheme. Six papers take this perspective.

Flores-Buills et al. and Sánchez-Gómez et al. propose the construction and validation of instruments designed to implement a QoL-enhancement approach among primary education students. Flores-Buills et al. present the development process as well as the reliability and validity evidence for RES-PRIM—a tool centered on students with and without NDD. This instrument aims to assess and promote resilience as a protective factor for QoL, subjective well-being, and participation, while also serving to guide interventions focused on improving these outcomes. Along the same lines, Sánchez-Gómez et al. present the adaptation and pilot study in Chile of the QoLI-PE. Originally developed in Spain (Grant No. PID2022-141048NA-I00), this instrument is designed to evaluate the QoL of students with intellectual and developmental disabilities as an input variable. By guiding educational interventions aimed at fostering QoL outcomes linked to access, participation, learning, and maximum personal development, the tool serves a dual purpose as both a pre- and post-intervention assessment to determine the impact of the provided supports.

Two studies highlight the significance of self-determination within this vision of QoL as a driving force. Shogren et al. examined the impact on the academic and social participation of autistic students of an intervention that combined the Self-Determined Learning Model of Instruction (SDLMI) and peer supports. In this regard, while QoL could be viewed as an *outcome* variable (reflected in academic and social participation), the authors conceptualize QoL as an overarching framework that must guide inclusive practices—serving as both an *input* and an *outcome*. Furthermore, the core intervention model (SDLMI) is anchored in the Causal Agency Theory of self-determination, a key guiding principle to mobilize QoL that centers the person with NDD in the decision-making process and forms the baseline for providing peer supports (as the throughput). Similarly, Arellano et al. present Life Story Work as an inclusive methodology to explore identity in ID. Although QoL was not directly measured in their study, developing a self-determined life project through the construction of one’s identity—essential for a person’s QoL—is a fundamental principle when working with this population. Within this framework, the methodology addressed in their study serves as the throughput to achieve this goal, enabling individuals to negotiate their identity, identify their strengths and goals, and resist stigma.

A similar “indirect” approach is proposed by Kum and Bilmez, who present a safety skills training program for children with ASD utilizing home-based modeling techniques. They argue that teaching these skills is essential to enhancing well-being and independence—both of which serve as a cornerstone of the contemporary paradigm of disability, thereby positioning QoL not merely as an outcome but as a core guiding principle. Finally, establishing a synthesis that integrates the neurodiversity paradigm with individual QoL models served as the *leitmotiv* for the work of Liñares-de-Marcos et al. In their mini-review, they presented the notion that QoL assessment must act as a driving force to ensure that supports—rather than aiming to “normalize the person”—serve as bridges for authentic and self-determined participation among the autistic population across all levels of support needs, while consistently respecting neurodivergent identity.

## Conclusions and moving forward

In this editorial, we have presented a synthesis of the articles published in the Research Topic “*Enhancing Quality of Life in Individuals with Neurodevelopmental Disorders: Pathways to Inclusion and Well-Being*,” organizing their contributions based on whether they address QoL as an outcome variable or as a driving force within logic models (input-throughput-outcome).

Based on this analysis, two conclusions emerge: (i) most papers (*n* = 10; 62.5%) conceptualize QoL as an outcome variable “reflecting” inclusion/participation; and (ii) among the six studies (37.5%) adopting the “alignment” approach, frameworks tend to be conceptual or indirect. That is, they define QoL through a logic model—as an input variable guiding the supports (the throughput) and an outcome variable evaluating the impact of actions. Notably, however, no study initiated from a baseline QoL assessment to align personal goals with tailored supports and measure subsequent impact. This gap points to a broader structural limitation in the field: while QoL is increasingly recognized as a guiding framework, it is rarely operationalized across the full input–throughput–outcome continuum in empirical research. This limitation is likely associated with the predominance of cross-sectional designs, the frequent reliance on proxy reports, and the scarcity of longitudinal and person-centered measurement strategies sensitive to intra-individual change. Consequently, current evidence tends to capture QoL as a reflective indicator of inclusion and participation rather than as a driver of personalized supports.

Accordingly, to advance the inclusion and well-being of people with NDD, it is essential to champion studies that provide empirical data from these comprehensive systems approaches. This is crucial for advancing evidence-based practices, thereby clarifying which supports, under what conditions, and starting from which personal aspirations, contribute to achieving the best possible personal outcomes.
